# Training pathways and formal curricula in robotic bariatric surgery: a systematic review

**DOI:** 10.1007/s11701-026-03247-2

**Published:** 2026-02-24

**Authors:** Francesco Brucchi, Daqi Zhang, Simona Bertoli, Santo Colosimo, Gianlorenzo Dionigi

**Affiliations:** 1 University of Milan, Via Festa del Perdono, 7, Milan, 20122 Italy; 2 Division of Thyroid Surgery, Jilin Provincial Key Laboratory of Surgical Translational Medicine, Jilin Provincial Precision Medicine Laboratory of Molecular Biology and Translational Medicine on Differentiated Thyroid Carcinoma, The China-Japan Union Hospital of Jilin University, Changchun, Jilin China; 3 Department of Food, Environmental and Nutritional Sciences (DeFENS), University of Milan, Milan, Italy; 4 Obesity Unit and Laboratory of Nutrition and Obesity Research, Department of Endocrine and Metabolic Diseases, IRCCS Istituto Auxologico Italiano, Milan, Italy; 5 Division of Surgery, Istituto Auxologico Italiano, Istituto di Ricovero e Cura a Carattere Scientifico (IRCCS), Milan, Italy; 6 Department of Pathophysiology and Transplantation, University of Milan, Milan, Italy

**Keywords:** Robotic bariatric surgery, Surgical education, Simulation-based training, Proficiency-based progression, Surgical curriculum

## Abstract

The adoption of robotic platforms in bariatric and metabolic surgery has increased steadily, raising important questions regarding how surgeons are trained to safely acquire robotic skills. While structured and competency-based training models are increasingly adopted in other fields of robotic surgery, training approaches in robotic bariatric surgery remain less standardized. A systematic review was conducted in accordance with PRISMA 2020 guidelines to identify studies describing structured training pathways or formal curricula for robotic bariatric surgery. PubMed, Embase, Scopus, and Cochrane Library, were searched from inception without date restrictions. Eligible studies explicitly reported training programs, curricula, or educational pathways for robotic bariatric procedures. Learning curve analyses without a defined curriculum were excluded. Data were synthesized using a structured narrative approach. Five studies met the inclusion criteria. Training models included stepwise intraoperative curricula, simulation-based and proficiency-driven programs, and modular educational interventions. Common components across curricula were simulation training, task decomposition, supervised progression, and defined competency benchmarks. Assessment strategies were heterogeneous and ranged from simulation-based proficiency thresholds to operative participation metrics and subjective workload measures. No study reported standardized certification or long-term competency outcomes. Structured training pathways for robotic bariatric surgery have been described and incorporate elements aimed at supporting safe skill acquisition. However, existing curricula remain heterogeneous and lack standardized assessment frameworks. Future efforts should focus on developing competency-driven and proficiency-based progression training models to support reproducible and safe adoption of robotic bariatric surgery.

**Supplementary Information:**

The online version contains supplementary material available at 10.1007/s11701-026-03247-2.

## Introduction

The adoption of robot-assisted surgery within bariatric and metabolic surgery has increased steadily over the past two decades, driven by technical advantages such as enhanced dexterity, improved ergonomics, and superior visualization in patients with complex body habitus [1, 2]. These features are particularly relevant for bariatric procedures, which often involve technically demanding dissection and suturing in anatomically constrained operative fields.

Despite this expansion, the integration of robotic technology into bariatric surgery raises important questions regarding training, skill acquisition, and patient safety. Bariatric procedures allow limited tolerance for error, and the introduction of robotic platforms requires surgeons to master a distinct set of console-based psychomotor and workflow-specific skills [3]. Traditional apprenticeship-based models, largely developed for open and laparoscopic surgery, may therefore be insufficient to support safe, cost-effective and efficient training in robotic bariatric practice.

In several other domains of robotic surgery, including urology, colorectal, and abdominal wall surgery, structured curricula and training pathways have increasingly been developed [4–8]. These programs often incorporate simulation, modular task decomposition, objective assessment metrics, and proficiency-based progression (PBP), shifting the focus from case numbers to demonstrated competence. Such approaches aim to standardize training, reduce variability in skill acquisition, and enhance patient safety during the learning process.

In contrast, training in robotic bariatric surgery remains less standardized, with heterogeneous approaches reported across institutions and training levels. While individual programs have described stepwise intraoperative teaching models, simulation-based curricula, or modular educational interventions, the existing evidence has not been systematically synthesized with a specific focus on formal training pathways and curricula.

The aim of this systematic review is to identify and synthesize structured training pathways and formal curricula for robotic bariatric surgery, with particular attention to their design, core components, target trainee populations, and assessment strategies. By contextualizing bariatric training within the broader evolution of robotic surgical education, this review seeks to clarify current practices and highlight opportunities for future curriculum development aligned with emerging competency-based frameworks.

## Methods

### Study design and reporting standards

This systematic review was conducted in accordance with the Preferred Reporting Items for Systematic Reviews and Meta-Analyses (PRISMA 2020) guidelines [9]. The research protocol was registered with the International Prospective Register of Systematic Reviews (PROSPERO) under registration number CRD420261288962 (http://www.crd.york.ac.uk/PROSPERO*).* A systematic search of the peer-reviewed literature published from January 1st, 2000, to January 25th, 2025, was conducted using the PubMed, Embase, Scopus, and Cochrane Library databases. A combination of keywords was used in the search: “education,” “simulation training,” “training,” “teaching,” “preceptorship,” “curriculum,” “robotic surgery,” “robotic surgical procedures,” “Bariatric Surgery,” “Obesity,” and “Metabolic Surgery,” “Robot-assisted Bariatric Surgery.” Search strategies for each database were developed using various combinations of keywords (detailed in Supplementary Materials, Fig. 1s). Additional references were identified by manually screening the bibliographies of retrieved articles, systematic reviews, and meta-analyses.

### Study selection

Two investigators (FB, SC) independently performed the literature search and data extraction using Rayyan systematic review software. They assessed the eligibility of all preliminarily identified records independently, first based on the title and then on the abstract. After the preliminary selection, the full-text manuscripts of relevant studies were carefully reviewed to confirm eligibility and to extract useful information. Any disagreements regarding eligibility were resolved by a third reviewer (GD). The study selection process is summarized using a PRISMA flow diagram.

### Eligibility criteria

#### Inclusion criteria

Studies were eligible for inclusion if they met all of the following criteria:

1. Population: surgeons, residents, fellows, or attending surgeons involved in robotic bariatric surgery.
2. Intervention: explicit description of a structured training pathway or formal curriculum for robotic bariatric surgery, including at least one of the following:

- modular or stepwise training models.
- simulation-based curricula (e.g., virtual reality, dry/wet lab, cadaveric training).
- structured mentorship or proctorship programs.
- credentialing or certification pathways.
- fellowship-based or institutionally defined robotic bariatric training programs.

3. Surgical approach: robot-assisted bariatric procedures.
4. Study type: original studies, including prospective or retrospective observational studies and educational program descriptions.
5. Language: articles published in English.

#### Exclusion criteria

Studies were excluded if they met any of the following criteria:

- learning curve analyses without an explicitly described training curriculum or pathway.
- studies reporting clinical or technical outcomes only, without an educational or training component.
- non-robotic bariatric surgery.
- case reports, narrative reviews, editorials, letters, or expert opinions.
- animal-only or laboratory studies not embedded within a defined training curriculum.

Learning curve studies were excluded unless they were clearly embedded within or explicitly linked to a structured training or educational program.

A formal curriculum was defined as a structured educational program with predefined components and progression criteria.

### Data extraction

Data extraction was performed using a predefined standardized form. The following variables were collected from each included study:

- study characteristics (year of publication, country, study design).
- target population (residents, fellows, attending surgeons).
- type of bariatric procedures included.
- structure and components of the training curriculum.
- use of simulation or modular training.
- mentorship, proctorship, or supervision models.
- assessment and competency metrics.
- credentialing or certification requirements, when reported.

### Quality assessment

The methodological quality of the included studies was evaluated using the Medical Education Research Study Quality Instrument (MERSQI) [10]. Two reviewers independently assessed the risk of bias across all eligible studies. Any discrepancies were resolved through consensus, and when necessary, by consultation with a third reviewer.

In addition, the selected articles were analyzed according to Kirkpatrick’s Evaluation Framework to assess the effectiveness, strengths, and limitations of the training programs [11]. This model examines four levels of educational impact: participants’ reactions, learning outcomes, behavioral changes in clinical practice, and effects on final outcomes. A comprehensive description of the MERSQI and Kirkpatrick frameworks, including their respective domains and scoring systems, is reported in Supplementary Table S1.

## Results

### Study selection

**Fig. 1:**
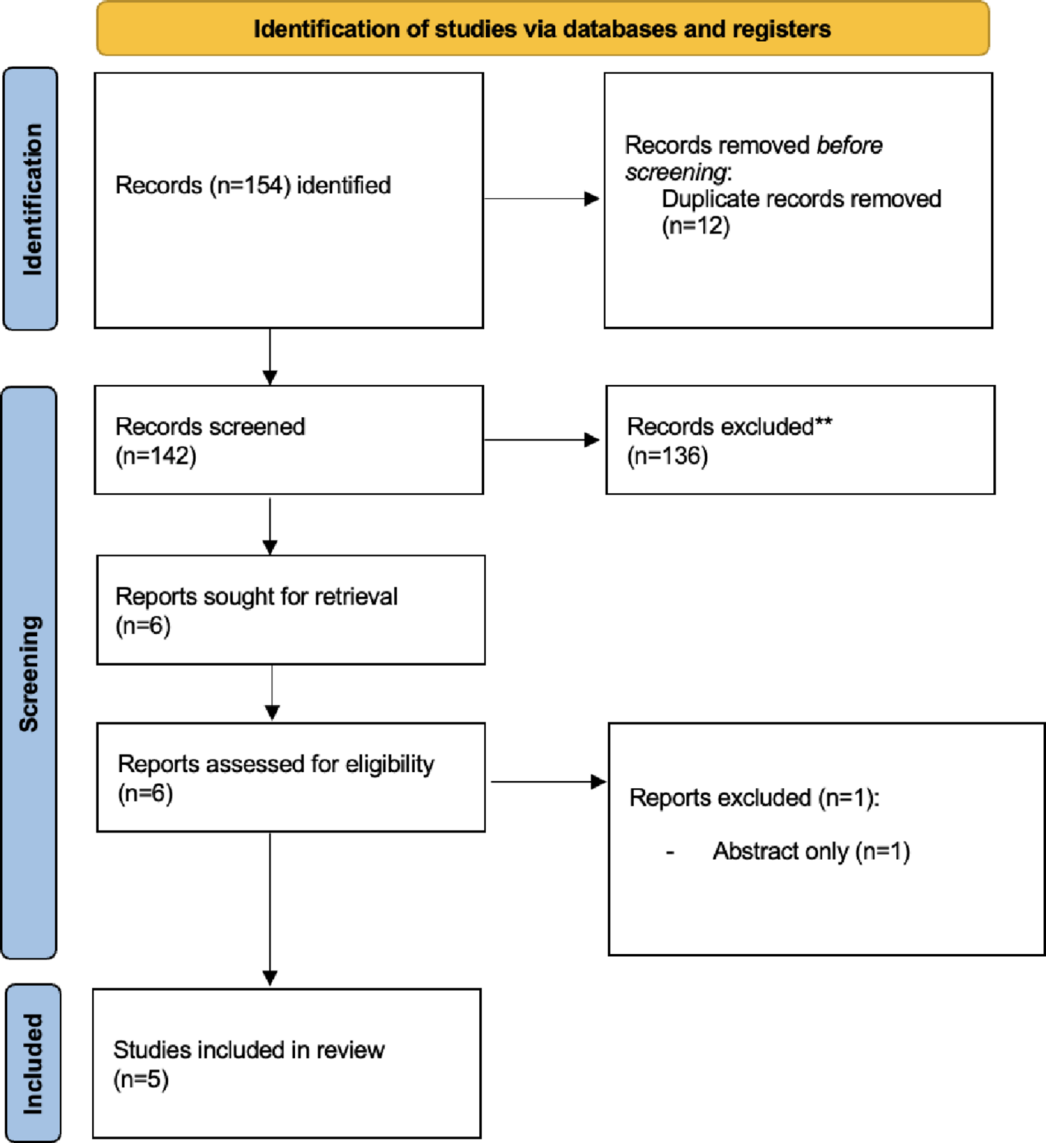
Flowchart of study screening according to PRISMA guidelines

The study selection process is illustrated in Fig. 1. The database search yielded 154 records. After removing 12 duplicates, 142 articles were screened based on titles and abstracts. Six full-text articles were subsequently assessed for eligibility. One study was excluded because of an incompatible study design, leaving five studies for inclusion in the final systematic review [12–16]. All included studies explicitly described structured training pathways or formal curricula for robotic bariatric surgery (Table 1).

**Table 1:** Characteristics of included studies on training pathways and curricula in robotic bariatric surgery

Author (Year)	Country	Study design	Enrollment years	MERSQI scale	Kirkpatrick level
Ali et al. (2007)	USA	Observational study	2003	10	3
Hashimoto et al. (2012)	USA	Retrospective study	2006–2010	10	3
Clanahan et al. (2024)	USA	Retrospective study	2020–2021	7.5	2
Gonçalves et al. (2024)	Portugal	Retrospective study	2024	6	1
Gomez et al. (2025)	USA	Randomized single-blinded study	NR	10	1

### Characteristics of included studies

The included studies were published between 2007 and 2025 and originated from academic institutions in USA and Europe. Study designs were predominantly observational or educational program evaluations, reflecting the nature of research in surgical education (Table 2).

**Table 2:** Characteristics of the training models or pathways and key findings of the included studies

Author (Year)	Target trainees	Bariatric procedure(s)	Training model	Key components	Assessment approach
Ali et al. (2007)	MIS fellows	RYGB	Stepwise intraoperative curriculum	Task decomposition, incremental responsibility, proctorship	Operative times, safety outcomes
Hashimoto et al. (2012)	Senior residents	Gastric banding	Stepwise proctored training model	Intraoperative stepwise responsibility, simulation orientation	Competency achievement, complications
Clanahan et al. (2024)	Residents (PGY1–5)	Sleeve, RYGB	Modular educational intervention	Annotated guides, narrated videos, dual-console training	Active console time metrics
Gonçalves et al. (2024)	Residents, surgeons	Sleeve, bypass (simulation)	Discovery course curriculum	Lectures, VR simulation, silicone models	Trainee questionnaires
Gomez et al. (2025)	Residents (PGY-3 or PGY-7)	Sleeve gastrectomy	Simulation-based training program	Dry-lab simulation, haptic feedback, staged exposure	NASA-TLX workload, operative metrics

Training programs targeted general surgery residents, minimally invasive surgery fellows, and attending surgeons transitioning to robotic bariatric practice. Specifically, Ali et al. [14] and Hashimoto et al. [12] focused primarily on resident- and fellow-level training, whereas Clanahan et al. [13], Gonçalves et al. [16], and Gomez et al. [15] included mixed cohorts of residents and attending surgeons.

Procedures addressed included robotic Roux-en-Y gastric bypass [14], robotic laparoscopic gastric banding [12], and robotic sleeve gastrectomy, either as a primary focus or within mixed bariatric cohorts [13, 15, 16].

### Types of training pathways and curricula

Three main categories of training models were identified across the included studies.

1. *Stepwise intraoperative training curricula*.

Early studies by Ali et al. [14] and Hashimoto et al. [12] described stepwise intraoperative training models, in which bariatric procedures were decomposed into discrete operative tasks. Trainees were progressively assigned increasing levels of responsibility under direct expert supervision, contingent on demonstrated competence. These curricula emphasized patient safety while enabling structured skill acquisition during live robotic bariatric surgery.

2. *Simulation-based curricula*.

Simulation-based training was a central component in several studies, although with varying objectives. Gomez et al. [15] described a simulation program incorporating haptic feedback and workload assessment, primarily aimed at evaluating trainee experience and cognitive load. Clanahan et al. [13] incorporated simulation and preparatory educational materials to support early console exposure. In contrast to stepwise intraoperative curricula, these programs focused predominantly on preoperative skill familiarization rather than progressive clinical autonomy.

3. *Modular educational programs and discovery courses*.

More recent studies by Gonçalves et al. [16] and Clanahan et al. [13] described modular educational programs combining didactic sessions, procedural videos, virtual reality simulation, and hands-on training using physical or synthetic models. These programs were designed as introductory or transitional pathways, particularly for trainees or surgeons with limited prior robotic experience, and were not directly linked to predefined operative competency milestones.

### Training components

Across the included studies, several core training components were consistently reported. Simulation training was used in four studies [12, 13, 15, 16], employing virtual reality platforms, dry-lab exercises, or procedure-specific simulators.

Stepwise task decomposition and supervised progression were central features in the curricula described by Ali et al. [14] and Hashimoto et al. [12], where trainees advanced through defined operative steps under expert proctorship. Mentorship and direct supervision were explicitly reported in these stepwise models and implicitly embedded within modular programs.

Defined competency benchmarks were inconsistently reported and were most clearly described in stepwise intraoperative curricula, while modular and simulation-focused programs generally lacked explicit progression thresholds.

### Assessment and evaluation strategies

Assessment methods varied substantially across studies. Ali et al. [14] and Hashimoto et al. [12] evaluated trainee progression through operative performance and increasing procedural responsibility in the operating room. Clanahan et al. [13] relied on measures of console exposure and participation, whereas Gomez et al. [15] focused on subjective workload and trainee experience during simulation. Gonçalves et al. [16] primarily assessed trainee reaction and satisfaction following educational courses.

Assessment strategies relied primarily on operative participation metrics, subjective workload measures, or progression in intraoperative responsibility, with no study using validated surgical performance scales such as OSATS or GEARS.

### Target population

Training pathways were directed at different stages of surgical training. Ali et al. [14] and Hashimoto et al. [12] primarily targeted general surgery residents and fellows, facilitating progressive integration into robotic bariatric procedures. Clanahan et al. [13], Gonçalves et al. [16], and Gomez et al. [15] included attending surgeons and mixed trainee cohorts, focusing on early exposure, familiarization with robotic platforms, and introductory skill development. Several programs were explicitly designed to support the safe integration of trainees into robotic bariatric surgery within high-volume academic centers.

### Methodological quality assessment (MERSQI)

Methodological quality, assessed using the Medical Education Research Study Quality Instrument (MERSQI), was overall low to moderate across the included studies (Supplementary Table S1). All studies were single-center investigations with predominantly observational or quasi-experimental designs. Ali et al. [14] and Hashimoto et al. [12] achieved the highest MERSQI scores, reflecting more structured study designs and the assessment of higher-level educational outcomes, including progressive intraoperative responsibility and behavioral change in the operating room.

Intermediate methodological quality was observed in Clanahan et al. [13], which evaluated targeted educational resources using objective participation metrics but lacked validated assessment instruments and long-term outcome measures. Lower MERSQI scores were assigned to Gonçalves et al. [16] and Gomez et al. [15], as these studies primarily relied on self-reported or reaction-level outcomes and did not assess behavioral change or downstream effects on clinical practice.

Across all included studies, formal validation of assessment instruments was inconsistently reported, and none evaluated long-term educational outcomes such as independent practice, credentialing, or certification. Overall, the MERSQI analysis highlights the predominantly descriptive nature of the existing literature on robotic bariatric training curricula and underscores the need for more rigorous, competency-oriented study designs.

## Discussion

To our knowledge, this is the first systematic review specifically examining training strategies in robotic bariatric surgery. Our findings indicate that, although structured training models have been described, existing curricula remain heterogeneous in design, scope, and assessment strategies, reflecting an evolving yet fragmented educational landscape.

### Evolution of training models in robotic bariatric surgery

The included studies illustrate a temporal evolution in training approaches. Early curricula were primarily based on stepwise intraoperative training, in which complex procedures—most notably robotic Roux-en-Y gastric bypass—were decomposed into discrete operative tasks progressively assigned under close supervision. These models emphasized patient safety and gradual autonomy and demonstrated that structured intraoperative teaching could be implemented without increasing perioperative risk [14].

More recent programs have increasingly incorporated simulation-based components, either as prerequisites for operative exposure or as structured adjuncts to clinical training. Procedure-specific simulation, modular educational resources, and preparatory materials were used to standardize baseline skills and facilitate early console participation [12, 13, 15, 16]. This shift reflects a transition from opportunistic learning toward more intentional curriculum design.

### Training components and educational strategies

Despite heterogeneity in implementation, several shared components emerged. Simulation—using virtual reality platforms, dry-lab exercises, or procedure-specific simulators—was consistently reported and aimed at mitigating early technical errors and cognitive workload [12, 13, 15, 16]. Stepwise task allocation and supervised progression further supported controlled skill acquisition, particularly in technically demanding bariatric procedures.

However, the depth of curricular structure varied substantially. While Ali et al. [14] and Hashimoto et al. [12] described progressive intraoperative pathways with increasing trainee responsibility, these programs lacked formal simulation requirements or predefined proficiency benchmarks. Other studies reported more limited interventions, such as preparatory resources [13], discovery courses [16], or standalone simulation focused on trainee experience [15]. Notably, none of the included studies described a fully integrated curriculum combining simulation, structured intraoperative progression, and formal proficiency benchmarks.

### Assessment and the gap in proficiency-based progression

A central finding of this review is the inconsistent use of objective assessment and competency benchmarks. Some studies employed explicit proficiency thresholds, particularly in simulation-based settings, whereas others relied on indirect indicators such as operative participation metrics or subjective workload measures. Importantly, no curriculum fully implemented a comprehensive proficiency-based progression (PBP) framework across the entire training pathway.

In other domains of robotic surgery, training paradigms increasingly emphasize competency-based advancement, validated performance metrics, and standardized assessment tools [4–6, 17]. By contrast, robotic bariatric surgery training appears to be at an earlier stage of this transition, with assessment strategies remaining largely local and heterogeneous. This gap may contribute to the persistent variability in training experiences across institutions.

### Target populations and transferability

The included curricula addressed a broad spectrum of learners, including residents, fellows, and attending surgeons transitioning to robotic practice. Although specific procedures varied, the underlying educational principles were largely transferable. This suggests that structured training pathways may be adaptable across bariatric procedures, provided that procedure-specific technical demands are adequately integrated.

#### Implications for future curriculum development

Our findings highlight several priorities for future development. First, greater standardization of curriculum structure, competency benchmarks, and assessment strategies is needed. Second, systematic integration of PBP principles may align robotic bariatric training with broader trends in surgical education, enhancing reproducibility and patient safety. Randomized evidence indicates that PBP training improves technical performance and reduces errors compared with conventional approaches [18, 19].

Third, the development of consensus-based curricula supported by professional societies and multicenter collaborations may facilitate scalable and externally validated training pathways. Such frameworks could support credentialing processes, quality assurance, and institutional governance, thereby promoting more consistent and transparent adoption of robotic bariatric surgery.

### Limitations

This review is limited by the small number of eligible studies and their predominantly observational design. Heterogeneity in training models and reported outcomes precluded quantitative synthesis. Long-term outcomes related to skill retention, certification, and independent practice were rarely reported. In addition, all included studies originated from high-resource settings, which may limit generalizability. Nevertheless, by focusing exclusively on explicitly described curricula, this review provides a focused and methodologically robust synthesis of current educational practices.

## Conclusion

Structured training pathways for robotic bariatric surgery have been described, including stepwise intraoperative models, simulation-based programs, and modular educational interventions. However, these curricula remain heterogeneous and lack standardized competency benchmarks. Future efforts should prioritize the development of integrated, proficiency-based, and consensus-driven training frameworks to support safe, reproducible, and accountable adoption of robotic bariatric surgery.

## Supplementary Information

Below is the link to the electronic supplementary material.

Supplementary Material 1

## Data Availability

All data generated or analyzed during this study are included in this published article and its supplementary material.

## Declarations

### PROSPERO ID

CRD420261288962.
