# Supplementary material for: Training pathways and formal curricula in robotic bariatric surgery: a systematic review
Source: J Robot Surg. 2026 Feb 24;20(1):286. doi: 10.1007/s11701-026-03247-2 (PMC12932264; doi:10.1007/s11701-026-03247-2)
Supplement: Supplementary file 1 — Supplementary Material 1 [file 11701_2026_3247_MOESM1_ESM.docx]

**Detailed MeSH Search**

(

("Bariatric Surgery"[Mesh]

OR bariatric surgery[tiab]

OR metabolic surgery[tiab]

OR robotic bariatric surgery[tiab]

OR robot-assisted bariatric surgery[tiab])

AND

("Robotic Surgical Procedures"[Mesh]

OR "Robotics"[Mesh]

OR robotic surgery[tiab]

OR robot-assisted surgery[tiab]

OR surgical robotics[tiab])

AND

("Education, Medical"[Mesh]

OR "Education, Medical, Graduate"[Mesh]

OR "Curriculum"[Mesh]

OR "Clinical Competence"[Mesh]

OR "Learning Curve"[Mesh]

OR "Simulation Training"[Mesh]

OR training pathway*[tiab]

OR training program*[tiab]

OR surgical training[tiab]

OR robotic training[tiab]

OR curriculum[tiab]

OR curricula[tiab]

OR fellowship[tiab]

OR credentialing[tiab]

OR certification[tiab]

OR mentorship[tiab]

OR proctorship[tiab]

OR competency-based training[tiab]

OR proficiency-based training[tiab]

OR structured training[tiab]

OR modular training[tiab])

)

**Table 1s: ​​overview of the MERSQI and Kirkpatrick assessment frameworks**

| **Tool** | **Purpose** | **Domains / Levels** | **Scoring** |
| --- | --- | --- | --- |
| **MERSQI** | Assess methodological quality of educational studies | 6 domains: Study Design, Sampling, Type of Data, Validity, Data Analysis, Outcomes | Score range: 5–18 |
| **Kirkpatrick Model** | Evaluate training effectiveness | Level 1: Reaction  Level 2: Learning  Level 3: Behavior  Level 4: Results | Descriptive (not scored) |

# **Supplementary Table S1. Methodological quality assessment using MERSQI**

| Study | Study Design (0–3) | Sampling (0–3) | Type of Data (0–3) | Validity of Evaluation Instrument (0–3) | Data Analysis (0–3) | Outcomes (0–3) | Total Score (0–18) |
| --- | --- | --- | --- | --- | --- | --- | --- |
| Ali et al. 2007 | 2.0 | 1.5 | 2.0 | 0.5 | 2.0 | 1.5 | 9.5 |
| Hashimoto et al. 2012 | 2.0 | 1.5 | 2.0 | 1.0 | 2.0 | 1.5 | 10.0 |
| Clanahan et al. 2024 | 1.5 | 1.0 | 2.0 | 0.5 | 1.5 | 1.0 | 7.5 |
| Gonçalves et al. 2024 | 1.0 | 1.0 | 1.5 | 0.5 | 1.0 | 1.0 | 6.0 |
| Gomez et al. 2025 | 2.0 | 1.5 | 2.0 | 1.0 | 2.0 | 1.5 | 10.0 |
